# Concurrent Treatment with Taxifolin and Cilostazol on the Lowering of β-Amyloid Accumulation and Neurotoxicity via the Suppression of P-JAK2/P-STAT3/NF-κB/BACE1 Signaling Pathways

**DOI:** 10.1371/journal.pone.0168286

**Published:** 2016-12-15

**Authors:** So Youn Park, Hae Young Kim, Hee Jeong Park, Hwa Kyoung Shin, Ki Whan Hong, Chi Dae Kim

**Affiliations:** 1 Department of Pharmacology, School of Medicine, Pusan National University, Gyeongsangnam-do, Republic of Korea; 2 Gene & Cell Therapy Research Center for Vessel-associated Diseases, Pusan National University, Gyeongsangnam-do, Republic of Korea; 3 Division of Meridian and Structural Medicine, Pusan National University, Gyeongsangnam-do, Republic of Korea; Massachusetts General Hospital, UNITED STATES

## Abstract

Taxifolin is a potent flavonoid that exerts anti-oxidative effect, and cilostazol increases intracellular cAMP levels by inhibiting phosphodiesterase 3 that shows antiinflammatory actions. BACE1 (β-site APP cleaving enzyme 1) is the rate-limiting enzyme responsible for the β-cleavage of amyloid precursor proteins to Aβ peptides. In this study, endogenous Aβ and C99 accumulation was explored in N2a Swe cells exposed to 1% FBS medium. Increased Aβ and C99 levels were significantly attenuated by taxifolin alone and in combination with cilostazol. Increased phosphorylated JAK2 at Tyr1007/1008 (P-JAK), phosphorylated STAT3 at Tyr 705 (P-STAT3) expressions and increased expressions of BACE1 mRNA and protein in the activated N2a Swe cells were significantly attenuated by taxifolin (10~50 μM), cilostazol (10~50 μM) alone and in combination at minimum concentrations. In these cells, decreased cytosol IκBα expression was elevated, and increased nuclear NF-κB p65 level and nuclear NF-κB p65 DNA binding activity were significantly reduced by taxifolin and cilostazol in a similar manner. Following STAT3 gene (~70% reduction) knockdown in N2a cells, Aβ-induced nuclear NF-κB and BACE1 expressions were not observed. Taxifolin, cilostazol, or resveratrol significantly stimulated SIRT1 protein expression. In SIRT1 gene-silenced (~50%) N2a cells, taxifolin, cilostazol, and resveratrol all failed to suppress Aβ_1-42_-stimulated P-STAT3 and BACE1 expression. Consequently, taxifolin and cilostazol were found to significantly decrease lipopolysaccharide (1–10 μg/ml)-induced iNOS and COX-2 expressions, and nitrite production in cultured BV-2 microglia cells and to increase N2a cell viability. In conclusion, taxifolin and cilostazol strongly inhibited amyloidogenesis in a synergistic manner by suppressing P-JAK2/P-STAT3-coupled NF-κB-linked BACE1 expression via the up-regulation of SIRT1.

## Introduction

Alzheimer’s disease (AD) is characterized by increased amyloid β (Aβ)-containing extracellular plaque and intracellular neurofibrillary tangles, which are associated with synaptic failure and cognitive deficits [1]. Enhanced amyloidogenic processing of amyloid precursor protein (APP) by β- and γ-secretase increases intracellular level of soluble oligomeric Aβ, which results in pronounced synaptic failure and eventually in memory decline [2,3]. Theoretically, Aβ accumulation can be reduced in AD patients by suppressing Aβ production or enhancing Aβ degradation and clearance. A membrane-associated C-terminal fragment of APP, C99, is liberated by the action of β-secretase, and this is subsequently cleaved by γ-secretase to produce Aβ peptide [4].

BACE1 (β-secretase, a membrane-bound aspartyl protease β-site APP cleaving enzyme 1) is a rate-limiting enzyme for β-amyloid production [5]. The expression of BACE1 protein and its activity have been demonstrated to be elevated in the brains of AD patients [6,7]. Buggia-Prevot et al. [8] proposed Aβ_1–42_ acts as a regulator of BACE1, and suggested exacerbated Aβ production modulates BACE1 promoter transactivation and its activity via an NF-κB-dependent pathway. Furthermore, Aβ has been shown to activate nuclear transcription factor NF-κB [9,10], which is activated during the early stages of AD, where RelA/p65 plays a critical role in neurons and astrocytes surrounding amyloid plaques in the brain, and elevates oxidative stress [11].

In addition, constitutive Janus kinase 2 (JAK2)/signal transducer and activator of transcription 1 (STAT1) signaling has been demonstrated to contribute to endogenous BACE1 expression and subsequent Aβ generation in neurons, and inhibition of the JAK2/STAT1 signaling pathway by AG490 (a JAK2 inhibitor) reduced the expression of endogenous BACE1 and Aβ production[12]. Grivennikov and Karin [13] postulated STAT3-mediated nuclear NF-κB activation plays an important role in the pathogenesis of cancer and neurodegenerative disease, despite the fact NF-κB is not the only transcription factor that cooperates with STAT3.

Taxifolin (dihydroquercetin, (2*R*,3*R*)-2-(3,4-dihydroxyphenyl)-3,5,7-trihydroxy-2,3-dihydrochromen-4-one) is a potent flavonoid found in onions, French maritime pine bark, and milk thistle [14], and has been shown to ameliorate cerebral ischemia-reperfusion injury in rats due to its anti-oxidative effect and to modulate NF-κB activation [15]. In addition, cilostazol (OPC-13013, 6-[4-(1-cyclohexyl-1H-tetrazol-5-yl) butoxy]-3,4-dihydro-2-(1H)-quinolinone) increases intracellular cAMP levels by inhibiting type 3 phosphodiesterase, and recently, was reported to improve memory impairment induced by an intracerebroventricular injection of Aβ_25–35_ in C57BL/6J mice [16].

Previous studies have shown [17,18], cilostazol inhibits proinflammatory factor (including remnant lipoprotein particle, TNFα and LPS)-stimulated increases in IκBα degradation and NF-κB p65 activity, and thus, suppresses the productions of inflammatory cytokines. Interestingly, Paris et al. [19] showed NF-κB inhibitors decreased both Aβ_1–40_ and Aβ_1–42_ production and suggested NF-κB inhibitors might be of therapeutic importance for the treatment of AD.

It is generally said that the currently available hitting-one-target drugs are insufficient for the treatment of AD, and thus, research designed to identify drug therapies that target multiple action sites is attracting more attention. Considering these points of view, combinatorial drug therapy that target multiple pathogenic factors are worthy of investigation for prevention and treatment of AD. For this reason, we investigated the efficacies of taxifolin alone and in combination with cilostazol with respect to the regulation of BACE1, Aβ accumulation, and the inhibition of neuroinflammation with a view towards possible intervention in Alzheimer’s disease.

In the present study, we examined the inhibitory effect of taxifolin and taxifolin plus cilostazol on the JAK2/STAT3/NF-κB signaling axis in Aβ production in N2a cells stably expressing human APP Swedish mutation (N2a Swe cells) with focus on the suppressive effects of taxifolin and of taxifolin plus cilostazol on the Aβ-induced mRNA and protein expressions of BACE1. In addition, we elucidated the mechanism(s) by which Aβ_1-42_-induced STAT3 and NF-κB activation are down-regulated by these drugs, and verified that taxifolin and cilostazol both inhibit P-STAT3, nuclear NF-κB p65, and BACE1 expression by upregulating SIRT1.

## Results

### Effects on Aβ-induced P-JAK2 and P-STAT3 expressions in activated N2a Swe cells

**Fig 1:**
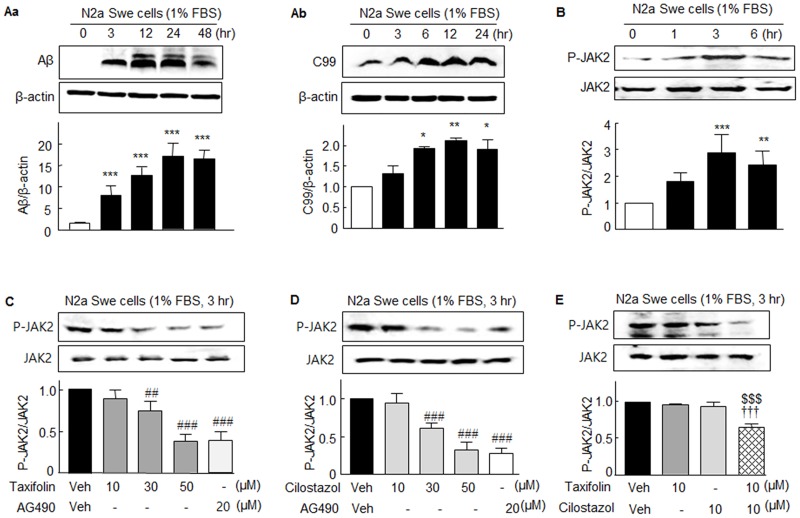
**Aa** and **Ab**. Increased intracellular accumulations of Aβ and C-terminal fragment β (C99) in N2a Swe cells. **B**. Time-dependent increases in the expression of JAK2 phosphorylated at Tyr1007/1008 (P-JAK2) in activated N2a Swe cells. **C** and **D**. Concentration-dependent decreases in P-JAK2 expression by taxifolin (10 ~ 50 μM), cilostazol (10 ~ 50 μM), and 20 μM AG490 (a JAK2 inhibitor) determined after culture in medium containing 1% FBS for 3 hr. JAK2 levels showed little change. **E**. Co-treatment with 10 μM taxifolin plus 10 μM cilostazol significantly decreased P-JAK2 expression. Results are the means ± SEM of 4–5 experiments. *P < 0.05, **P < 0.01, ***P < 0.001 vs. Zero time; ^##^*P* < 0.01, ^###^*P* < 0.001 vs. Vehicle (Veh); ^$ $ $^*P* < 0.001 vs. 10 μM Cilostazol alone; ^†††^ *P* < 0.001 vs. 10 μM Taxifolin alone.

Initially, we sought to confirm the endogenous production of Aβ is accompanied by increases in the expressions of P-JAK2 and P-STAT3 in N2a Swe cells [20]. As shown in Fig 1Aa and 1Ab, activated N2a Swe cells upon exposure to 1% FBS revealed a large accumulation of intracellular Aβ (4 kDa) and an elevated C-terminal fragment β (C99) level (11 kDa) in a time (3–48 hr)-dependent manner with maximum values at 12 ~ 24 hr (Fig 1Aa and 1Ab). After obtaining this confirmation, we assessed the expressions of P-JAK2 and P-STAT3 in activated N2a Swe cells.

It has been reported constitutive JAK2/STAT1 activation mediates endogenous BACE1 expression in neurons, and that inhibition of JAK2/STAT1 signaling reduces basal levels of BACE1 expression and Aβ generation [12]. When N2a Swe cells were cultured for 1, 3, or 6 hr in medium containing 1% FBS, phosphorylated JAK2 at Tyr1007/1008 (P-JAK2) expression was significantly elevated at 3 hr (2.89 ± 0.68 fold, *P* < 0.001) and then declined at 6 hr (2.43 ± 0.51 fold; *F*_3,8_ = 12.88, *P* < 0.0005) (Fig 1B). However, increased P-JAK2 expression determined at 3 hr in medium containing 1% FBS was concentration-dependently decreased by taxifolin (10 ~ 50 μM; *F*_3,12_ = 39.40, *P* < 0.0001), by cilostazol (10 ~ 50 μM; *F*_3,12_ = 47.02, *P* < 0.0001), and by 20 μM AG490 (a JAK2 inhibitor) (Fig 1C & 1D). However, JAK2 levels were little changed. Intriguingly, the expression of P-JAK2 was not affected by 10 μM taxifolin or 10 μM cilostazol, but was significantly attenuated by co-treatment with 10 μM of taxifolin plus 10 μM cilostazol (to 0.65 ± 0.05 fold, *P* < 0.001, N = 5) (Fig 1E).

**Fig 2:**
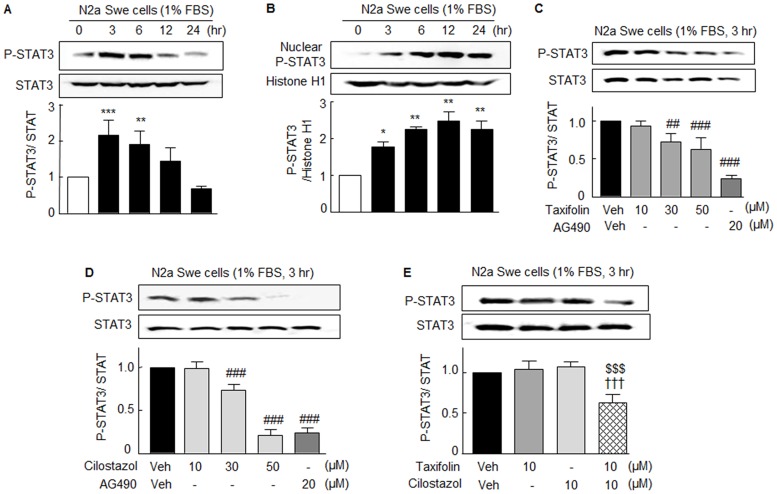
Aβ-induced P-STAT3 and STAT3 protein expressions in N2a Swe cells and their reductions by taxifolin and cilostazol. Time-dependent changes in the expression of STAT3 phosphorylated at Tyr705 (P-STAT3) in cytosolic (**A**) and nuclear (**B**) fractions of activated N2a Swe cells (3 hr after 1% FBS). **C** and **D**. Concentration-dependent decreases in elevated P-STAT3 expression induced by taxifolin (10 ~ 50 μM), cilostazol (10, 30, 50 μM), and 20 μM AG490. **E**. Co-treatment with 10 μM taxifolin plus 10 μM cilostazol significantly suppressed P-STAT3 expression. Results are the means ± SEMs of 4–7 experiments. *P < 0.05, **P < 0.01, ***P < 0.001 vs. Zero time; ^##^*P* < 0.01, ^###^*P* < 0.001 vs. Vehicle (Veh); ^$ $ $^*P* < 0.001 vs. Cilostazol alone; ^†††^ *P* < 0.001 vs. 10 μM Taxifolin alone.

In line of P-JAK2 expression, when N2a Swe cells were exposed to depleted FBS in culture medium, the expression of P-STAT3 at Tyr 705 (P-STAT3) in cytosol was significantly elevated at 3 hr (2.14 ± 0.42 fold, *P* < 0.001) and then declined (*F*_4,15_ = 16.63, *P* < 0.0001) (Fig 2A), which posed the question: Where did P-STAT3 move to? Thus, we investigated the time-dependent nuclear translocation of P-STAT3. As shown in Fig 2B, the expression of nuclear P-STAT3 was significantly elevated from 3 to 12 hr, suggesting nuclear translocation of P-STAT3. Increased nuclear P-STAT3 levels were decreased by taxifolin (10 ~ 50 μM; *F*_3,12_ = 12.66, *P* < 0.0005), cilostazol (10, 30, 50 μM; *F*_3,8_ = 136.2, *P* < 0.0001), and by 20 μM AG490 (p<0.05), suggesting P-STAT3 expression was mediated via P-JAK2 activation (Fig 2B and 2C). As was observed for P-JAK2 expression, P-STAT3 expression was not influenced by 10 μM taxifolin or 10 μM cilostazol. However, treatment with 10 μM taxifolin plus 10 μM cilostazol synergistically suppressed P-STAT3 expression to 0.63 ± 0.10 fold (*P* < 0.001, N = 7) (Fig 2D).

### Effects on Aβ-induced IκBα and NF-κB p65 expressions in the activated N2a Swe cells

**Fig 3:**
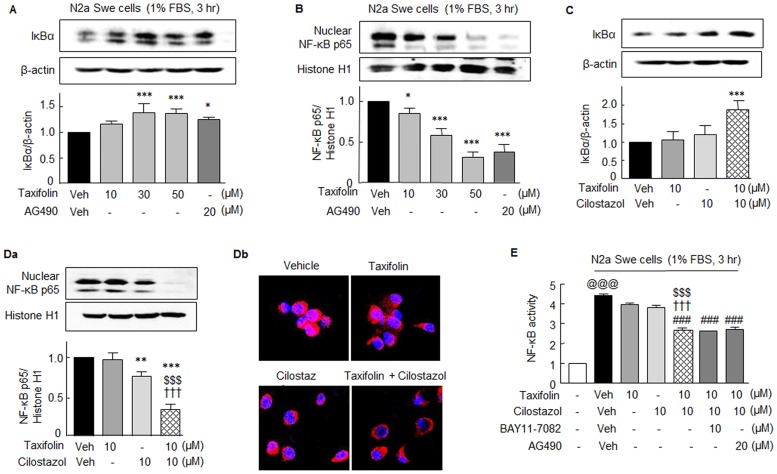
Aβ-induced changes in the expressions of IκBα and NF-κB p65, and effects of taxifolin and cilostazol in N2a Swe cells. Concentration-dependent increases in IκBα in cytosol (**A**) and decreases in NF-κB in nuclei (**B**) by taxifolin in activated N2a Swe cells (3 hr after 1% FBS) as compared with 20 μM AG490. Co-treatment with 10 μM taxifolin and 10 μM cilostazol significantly increased IκBα levels in cytosol (**C**) and decreased NF-κB levels in nucleus (**Da**), which was confirmed by immunofluorescence study (**Db**). **E**. Significant decrease in DNA binding activity of NF-κB by co-treatment with 10 μM taxifolin and 10 μM cilostazol as compared with taxifolin or cilostazol monotherapy. Results are the means ± SEMs of 4 experiments. *P < 0.05, **P < 0.01, ***P < 0.001 vs. Vehicle (Veh); ^$ $ $^*P* < 0.001 vs. Cilostazol alone; ^†††^ *P* < 0.001 vs. 10 μM Taxifolin alone; ^###^ *P* < 0.001 vs. Vehicle (Veh); ^@@@^*P* < 0.001 vs. None.

Many studies have documented a close correlation between the extent of NF-κB activation and Aβ peptide levels [19]. We previously found that cilostazol strongly inhibits NF-κB activation [17,18]. Herein, we assessed the impact of taxifolin on the Aβ-stimulated degradation of IκBα in cytosol and on the nuclear translocation of NF-κB p65. When N2a Swe cells were cultured for 3 hr in the medium containing 1% FBS, IκBα expression was decreased in cytosol, and this was significantly elevated by adding 30 μM of taxifolin to 1.39 ± 0.18 fold (*P* < 0.001), and by 20 μM AG490 to 1.26 ± 0.04 fold (*P* < 0.05) (Fig 3A). In line with these observations, nuclear NF-κB p65 levels were strikingly elevated at 3 hr after culture in the medium containing 1% FBS, and this increase in nuclear NF-κB p65 was concentration-dependently reduced by pretreating 30 μM taxifolin to 0.58 ± 0.08 fold (*P* < 0.001), and by pretreating 20 μM AG490 to 0.38 ± 0.09 fold (*P* < 0.001) (Fig 3B).

As was observed for P-JAK2 and P-STAT3 expressions, decreased IκBα expression in the cytosol of activated N2a Swe cells (1% FBS at 3 hr) was little altered by pretreating 10 μM taxifolin or 10 μM cilostazol. However, co-treatment with 10 μM taxifolin and 10 μM cilostazol strikingly elevated IκBα expression to 1.89 ± 0.24 fold (*P* < 0.001) (Fig 3C). Nuclear NF-κB p65 levels were not changed by 10 μM taxifolin, but were attenuated by 10 μM cilostazol to 0.76 ± 0.06 fold (*P* < 0.01). Intriguingly, treatment with 10 μM taxifolin plus 10 μM cilostazol, markedly decreased nuclear NF-κB p65 expression to 0.34 ± 0.07 fold (*P* < 0.001) (Fig 3Da). These findings were confirmed by immunofluorescence study (Fig 3Db). These results support the synergistic effect of combinatorial taxifolin and cilostazol therapy.

NF-κB p65 DNA binding assays revealed a large increase in NF-κB activity in activated N2a Swe cells (1% FBS at 3 hr), and this was significantly decreased by pretreatment with 10 μM taxifolin plus 10 μM cilostazol (*P* < 0.001), whereas the respective monotherapy had only marginal effects. These reductions in DNA binding activity were verified by pretreating cells with Bay11-7082 (10 μM; an inhibitor of IκBα phosphorylation [21]) and AG490 (10 μM, a JAK2 inhibitor) (Fig 3E), indicating changes in NF-κB activity are closely linked to the JAK2/STAT3 signaling pathways.

### Effects on Aβ-induced BACE1 mRNA and protein expressions in N2a Swe cells

Shimmyo et al. [22] reported natural flavonoids, such as, myricetin, quercetin, and kaempferol, directly inhibit BACE1 enzyme activity in a concentration-dependent manner and concomitantly inhibit Aβ_1–42_ levels. As many of the flavonoids tested inhibited β-cleavage of APP, we sought to determine whether treatments with taxifolin or taxifolin plus cilostazol directly inhibit β-secretase (BACE1) protein.

**Fig 4:**
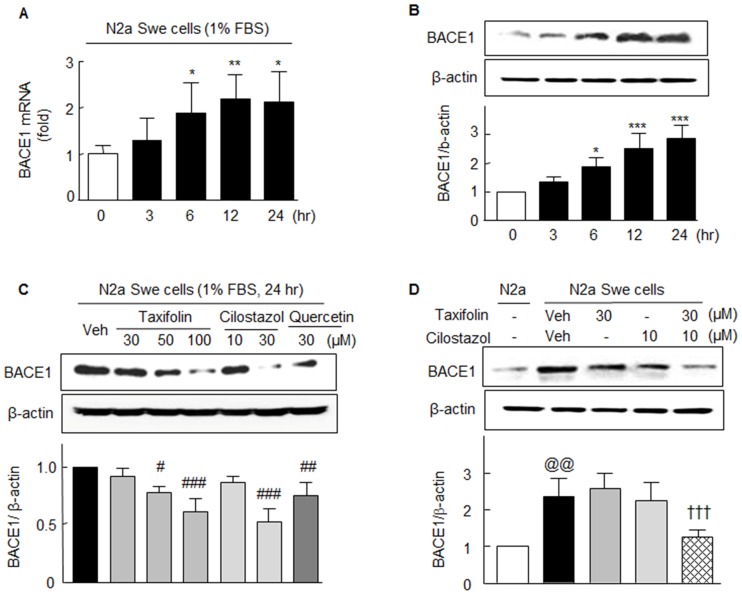
Aβ-induced BACE1 mRNA and protein expressions in activated N2a Swe cells and their reductions by taxifolin and cilostazol. Time (3 ~ 24 hr)-dependent changes in BACE1 mRNA (**A**) and protein (**B**) expressions. **C**. Significant decreases in BACE1 protein expression by taxifolin (50 or 100 μM), cilostazol (30 μM), and by 30 μM quercetin (a flavonoid) after 24 hr of treatment. **D**. Synergistic suppression of BACE1 expression by 30 μM of taxifolin plus 10 μM cilostazol as compared with the suppressions induced by monotherapies. Results are the means ± SEMs of 4 experiments. *P < 0.05, **P < 0.01, ***P < 0.001 vs. Zero time; ^#^*P* < 0.05, ^##^*P* < 0.01, ^###^*P* < 0.001 vs. Vehicle (Veh); ^@@^ *P* < 0.01 vs. N2a cells; ^†††^ *P* < 0.001 vs. Vehicle (Veh) of N2a Swe cells.

Upon exposing N2a Swe cells to medium containing 1% FBS, BACE1 mRNA expression significantly and time-dependently increased (from 6 ~ 24 h; *F*_4,15_ = 3.92, *P* < 0.022), as did BACE1 protein expression (*F*_4,15_ = 20.74, *P* < 0.0001), and both peaked at 12 to 24 hr (Fig 4A and 4B). Furthermore, increased BACE1 protein expression determined at 24 hr was significantly inhibited by taxifolin (50 and 100 μM), 30 μM cilostazol, or 30 μM quercetin (a flavonoid) (Fig 4C). Intriguingly, co-treatment with 30 μM taxifolin plus 10 μM cilostazol synergistically suppressed BACE1 expression (from 2.36 ± 0.49 fold to 1.26 ± 0.20 fold, *P* < 0.01), whereas the increased BACE1 expression was not attenuated by 30 μM taxifolin or 10 μM cilostazol alone (Fig 4D).

### Effects of drugs on STAT3-gene knockdown cells

**Fig 5:**
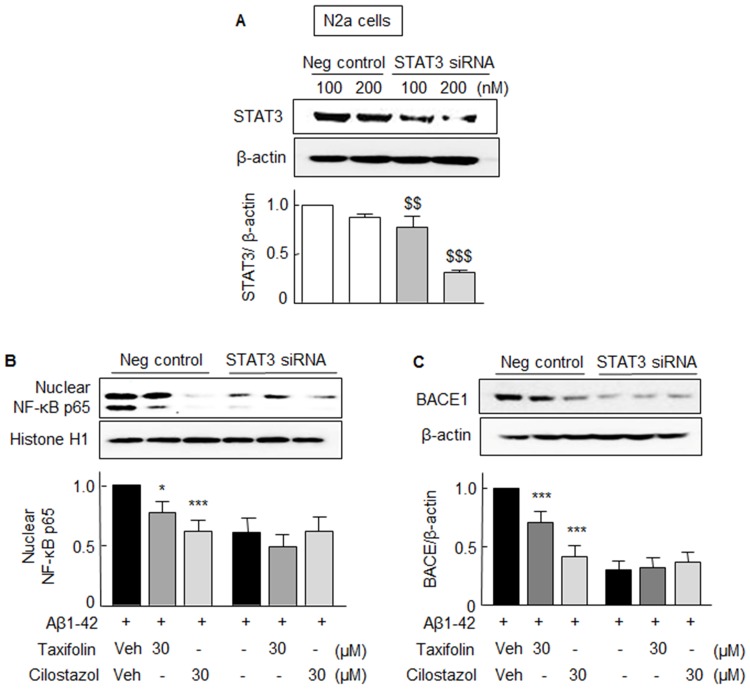
**A**. Analysis of the effects of STAT3-knockdown in N2a cells. In N2a cells transfected with 200 nM of STAT3 siRNA, STAT3 protein levels were reduced to ~ 30% of the level of negative controls. **B**. In STAT3 gene-silenced cells, Aβ_1–42_ did not elevate NF-κB p65 (**B**) or BACE1 levels (**C**), whereas it did so in cells transfected with scrambled siRNA duplex (negative control). Results are the means ± SEMs of 4 experiments. ^$ $ $^*P* < 0.01, ^$ $ $^*P* < 0.001 vs. Negative control; *P < 0.05, ***P < 0.001 vs. Vehicle (Veh).

To confirm that the Aβ _1-42_-induced NF-κB p65 and BACE1 expressions were mediated via P-STAT3 activation, N2a cells were transfected with STAT3 siRNA (200 nM). After silencing the STAT3 gene (~70% reduction in STAT3 protein expression, Fig 5A), Aβ-induced nuclear NF-κB p65 and BACE1 expressions were not observed (Fig 5B and 5C). However, in cells transfected with scrambled siRNA duplex (negative control), taxifolin (30 μM) or cilostazol (30 μM) significantly suppressed the expressions of nuclear NF-κB p65 and BACE1. These results indicate Aβ _1-42_-induced increases in the expressions of NF-κB p65 and BACE1 are dependent on STAT3 activation.

### Failure to suppress Aβ_1-42_-induced P-STAT3 and BACE1 in SIRT1 knockdown cells

**Fig 6:**
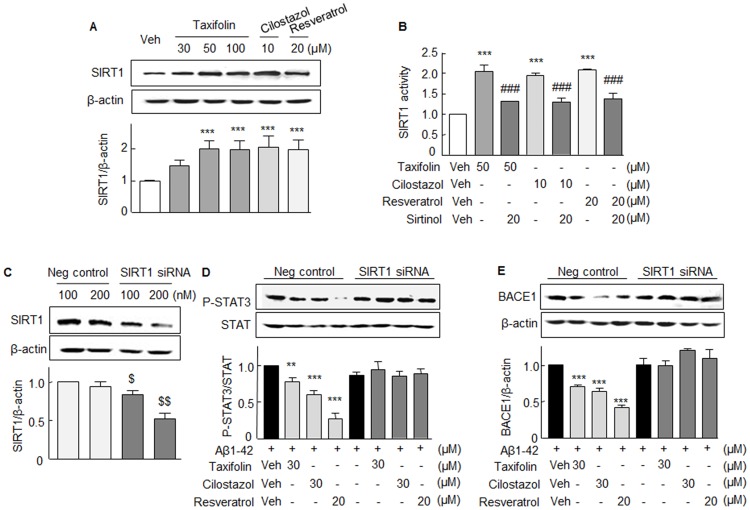
Stimulation of SIRT1 protein expression (**A**) and SIRT1 deacetylase activity in the absence and presence of sirtinol (**B**) by taxifolin (TFL), cilostazol (CSZ), and resveratrol (RES) in N2a cells. **C**. Analysis of the effects of SIRT1gene-knockdown in N2a cells. In N2a cells subjected to SIRT1 siRNA-gene silencing, SIRT1 protein expression reduced to ~50% by 200 nM SIRT1 siRNA. **D** and **E**. Failure of SIRT1 gene-silenced cells to inhibit Aβ_1-42_-stimulated P-STAT3 and BACE1 expressions as compared with the inhibitions observed in scrambled siRNA duplex (negative control) transfected cells. Results are the means ± SEMs of 4 experiments. **P < 0.01, ***P < 0.001 vs. Vehicle (Veh); ^###^*P* < 0.001 vs. Absence of sirtinol; ^$^*P* < 0.05, ^$ $ $^*P* < 0.01 vs. Negative control.

In a previous study [21], we observed cilostazol (3–10 μM) stimulated SIRT1 mRNA expression in N2a cells, and thus, in the present study, we examined whether taxifolin could stimulate SIRT1 protein expression in N2a cells. As shown in Fig 6A, taxifolin (30, 50, 100 μM), cilostazol (10 μM), and resveratrol (20 μM, a SIRT1 activator) all significantly stimulated SIRT1 protein expression. In addition, SIRT1 deacetylase activity was strikingly increased by 50 μM taxifolin, 10 μM cilostazol, and 20 μM resveratrol, and these increases were significantly attenuated by pretreatment with 20 μM sirtinol (SIRT1 inhibitor) (Fig 6B). Thus, we investigated whether taxifolin or cilostazol-induced inhibitions of Aβ_1-42_- upregulated P-STAT3 and BACE1 expressions were mediated via the activation of SIRT1. Accordingly, N2a cells were transfected with SIRT1 siRNA or scrambled siRNA duplex (negative control). In SIRT1 siRNA transfected N2a cells, SIRT1 protein expression was reduced to ~50% by 200 nM SIRT1 siRNA (Fig 6C). In the absence of Aβ_1-42,_ expression of P-STAT3 and BACE1 was not influenced by taxifolin and cilostazol (Data not shown).

In SIRT1 gene-silenced cells, 30 μM taxifolin 30 μM, 30 μM cilostazol, and 20 μM resveratrol all failed to suppress Aβ_1-42_-stimulated expressions of P-STAT3 and BACE1, whereas in cells transfected with scrambled siRNA duplex (negative control), 30 μM taxifolin, and 30 μM cilostazol significantly suppressed the expressions of P-STAT3 and BACE1 expression (Fig 6D and 6E). These results indicate that both taxifolin and cilostazol inhibit the expressions of P-STAT3 and BACE1 by up-regulating SIRT1.

### Synergistic inhibitory effects of combined treatment with taxifolin and cilostazol on pro-inflammatory factors in BV-2 cells

Compelling evidence suggests that Aβ deposition in AD is associated with marked inflammatory response due to microglia activation, and that neuronal cell death occurs as a consequence of uncontrolled neuro-inflammatory responses [23,24]. In the present study, we examined the protective effects of taxifolin, cilostazol, and taxifolin plus cilostazol on the LPS-induced activation of BV-2 cells by examining iNOS and COX-2 expressions and nitrite levels as markers of neuroinflammation.

**Fig 7:**
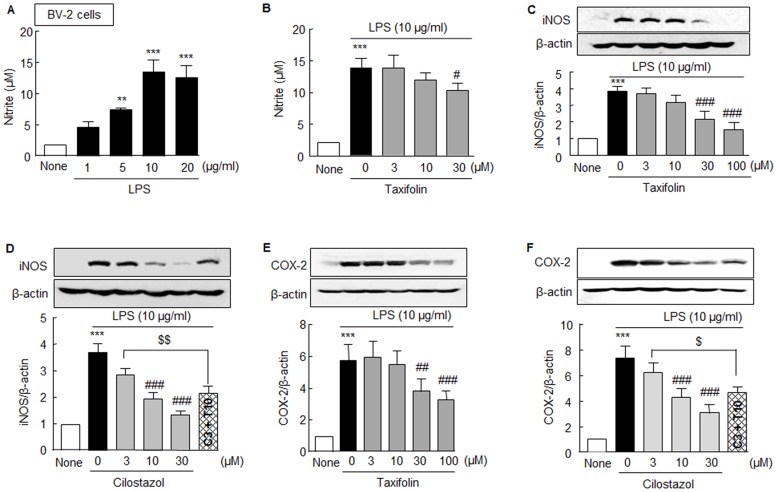
Inhibitory effects of taxifolin, cilostazol, and of taxifolin plus cilostazol on LPS-induced iNOS, COX-2 expressions, and nitrite levels in BV-2 microglia cells. **A**. Concentration-dependent LPS (1 ~ 20 μg/ml)-induced increase in nitrite formation. **B**. Inhibitory effect of taxifolin (3, 10, 30 μM) on LPS-stimulated nitrite levels. **C** and **D**. Concentration-dependent inhibition of taxifolin (3, 10, 30 or 100 μM) and cilostazol (3, 10, or 30 μM) on LPS (10 μg/ml)-stimulated iNOS expression, and synergistic inhibition of iNOS by 3 μM cilostazol (C3) plus 10 μM taxifolin (T10). **E**. Inhibitory effect of taxifolin (3, 10, 30 or 100 μM) on LPS-stimulated COX-2 expression. **F**. Inhibitory effect of cilostazol (3, 10, or 30 μM) on LPS-stimulated COX-2 expression, and of 3 μM cilostazol (C3) plus 10 μM taxifolin (T10) on the inhibition of COX2. Results are the means ± SEMs of 4–6 experiments. **P < 0.01, ***P < 0.001 vs. None; ^#^*P* < 0.05, ^##^*P* < 0.01, ^###^*P* < 0.001 vs. Vehicle; ^$^*P* < 0.05; ^$ $ $^*P* < 0.01 vs. 3 μM of Cilostazol.

Upon exposure of BV-2 microglial cells to LPS, nitrite (a surrogate of NO production) levels significantly increased in a concentration-dependent manner (1 ~ 20 μg/ml, *F*_4,10_ = 44.09, *P* < 0.0001) (Fig 7A). LPS (10 μg/ml)-induced nitrite increases (13.8 ± 1.7 μM) were diminished by 30 μM taxifolin to 10.4 ± 1.1 μM (*F*_3,9_ = 37.20, *P* < 0.0001) (Fig 7B). In line with these results, the up-regulation of iNOS induced by LPS (10 μg/ml) was concentration-dependently attenuated by taxifolin (3 ~ 100 μM, *F*_4,15_ = 25.60, *P* < 0.0001) (Fig 7C) and by cilostazol (3 ~ 30 μM, *F*_3,12_ = 48.65, *P* < 0.0001). Interestingly, LPS (10 μg/ml)-induced iNOS expression (3.68 ± 0.34 fold) was further diminished to 2.14 ± 0.27 fold (*P* < 0.01) by treatment with 3 μM cilostazol plus 10 μM taxifolin, whereas treatments with taxifolin or cilostazol alone did not attenuate LPS-induced iNOS expression at all (Fig 7D).

In addition, increased COX-2 expression by LPS (10 μg/ml) was also concentration-dependently diminished by taxifolin (3 ~100 μM, *F*_4,25_ = 12.74, *P* < 0.0001) and by cilostazol (3 ~ 30 μM, *F*_3, 12_ = 26.83, *P* < 0.0001), and LPS (10 μg/ml)-induced COX-2 expression (7.40 ± 0.90 fold) was significantly attenuated to 4.66 ± 0.47 fold (*P* < 0.05) by treatment with 3 μM cilostazol plus 10 μM taxifolin, whereas treatments with taxifolin or cilostazol alone did not decrease LPS-induced COX-2 expression (Fig 7E and 7F). These results indicate that co-treatment with taxifolin and cilostazol exerts anti-inflammatory effects in a synergistic manner.

### Inhibitory effects on Aβ and C-terminal fragment β (C99) accumulations in activated N2a Swe cells

It has been reported BACE1 knock-out mice lack the ability to produce Aβ [25]. Furthermore, C-terminal fragment (CTF) β (C99) is the earliest β-APP catabolite and a main contributor to intracellular β-APP-related immunoreactivity [26], and has been reported to be neurotoxic due to its ability to form large insoluble aggregates, and thus, C99 is considered to play a key role in the pathogenesis of AD [27].

**Fig 8:**
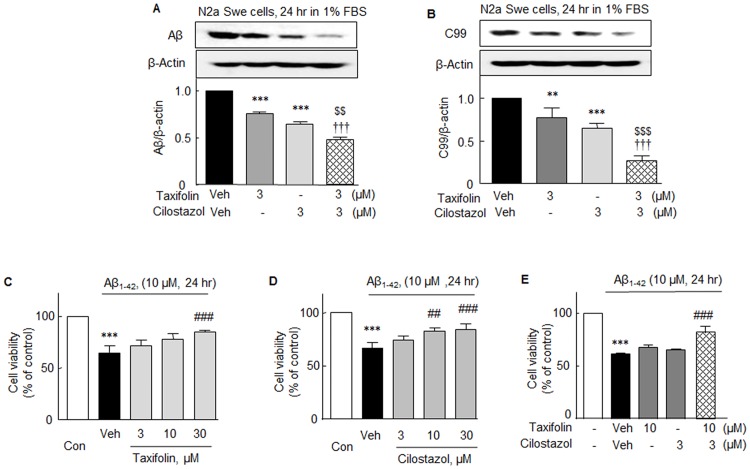
Upper panel: Inhibitory effects of taxifolin, cilostazol, and of taxifolin plus cilostazol on accumulations of intracellular Aβ (**A**) and C99 (**B**) in activated N2a Swe cells. Results are the means ± SEMs of 4 experiments. **P < 0.01, ***P < 0.001 vs. Vehicle (Veh); ^$ $ $^*P* < 0.01, ^$ $ $^*P* < 0.001 vs. 3 μM Cilostazol; ^†††^*P* < 0.001 vs. 3 μM Taxifolin. Lower Panel: Effects of taxifolin (**C**), cilostazol (**D**), and of taxifolin plus cilostazol **(E)** on Aβ_1-42_-induced cytotoxicity. Cell viabilities were assessed using an MTT assay. Aβ_1–42_ monomer (10 μM) was incubated without and with taxifolin and/or cilostazol at 37°C for 24 h and cell viability was measured. The results shown represent 4 independent experiments and are presented as the means ± SEMs of percentages versus control values. ***P < 0.001 vs. Control. ^##^*P* < 0.01; ^###^*P* < 0.001 vs. Vehicle (Veh).

As mentioned in Fig 1Aa and 1Ab, time-dependent increases in the endogenous accumulations of Aβ and C99 were observed in activated N2a Swe cells. In this study, the increase in Aβ level (at 24 hr) was significantly decreased by 3 μM of taxifolin or 3 μM of cilostazol to 0.76 ± 0.02 fold (*P* < 0.001) and 0.65 ± 0.01 fold (*P* < 0.001), respectively. When taxifolin and cilostazol co-administered, Aβ levels fell more markedly (to 0.48 ± 0.03 fold) as compared with taxifolin (*P* < 0.001) or cilostazol (*P* < 0.001) monotherapy (Fig 8A). Similarly, endogenous accumulations of C99 level observed at 24 hr also significantly decreased after treatment with 3 μM taxifolin or 3 μM cilostazol, and decreased more prominently to 0.27 ± 0.06 fold (*P* < 0.001) when they were co-treated (Fig 8B).

### Enhanced cell viability by combination therapy

In the present study, an MTT assay was used to obtain information on N2a neuronal cell cytotoxicity and cell viability. Exposure of N2a cells to 10 μM of Aβ_1–42_ for 24 h resulted in a significant decline in cell viability to 65.46 ± 6.24% (*P* < 0.001). The addition of 30 μM taxifolin to culture medium significantly reduced this decrease to 84.71 ± 2.05% (*P* < 0.001), but at 10 μM taxifolin had little effect (Fig 8C). Treatment with 10 or 30 μM of cilostazol also significantly increased cell viability from 66.64 ± 5.29% to 79.18 ± 2.38% (*P* < 0.01) and to 88.84 ± 2.38% (*P* < 0.001), respectively, whereas treatment with 3 μM cilostazol had little effect (Fig 8D). Intriguingly, decreased cell viability induced by 10 μM of Aβ_1–42_ (61.37 ± 1.0%) was not affected by 10 μM taxifolin or 3μM cilostazol, but was significantly recovered by 10 μM taxifolin plus 0.3 μM cilostazol to 81.99 ± 5.73 (*P* < 0.001) (Fig 8E). These results strongly suggest that the combination therapy with taxifolin plus cilostazol exerts a cytoprotective effect in a synergistic manner.

## Discussion

The present study shows that endogenously accumulated Aβ and C-terminal fragment β (C99) liberated from activated N2a Swe cells markedly increased the expressions of P-JAK, P-STAT3 and enhanced the nuclear translocation of NF-κB p65 and its activity, and that these events subsequently resulted in significant increases in the mRNA and protein expressions of BACE1 and amyloidogenesis. Taxifolin and cilostazol when combined even in lower concentrations strongly inhibited amyloidogenesis in a synergistic manner by suppressing P-JAK2/P-STAT3-coupled, NF-κB-linked, BACE1 expression via the up-regulation of SIRT1.

Recently, we showed that endogenously accumulated Aβ in N2a Swe cells (expressing human APP Swedish mutation) was significantly and concentration-dependently diminished by cilostazol. SIRT1-linked retinoic acid receptor-β activation by cilostazol underlies its pharmacological actions with respect to increasing ADAM10/α-secretase activity and reducing intracellular accumulations of full-length-APP and Aβ [28]. The present study highlights the synergistic effects of co-treatment with taxifolin plus cilostazol at low concentrations in terms of lowering of the intracellular accumulations of Aβ and C99 by suppressing BACE1 expression and reducing the generations of proinflammatory substances.

It is noteworthy that BACE1 gene promoter contains a NF-κB binding site [8]. Chen et al. [29] have reported both BACE1 and NF-κB p65 levels significantly increased in the brains of AD patients, and that increased NF-κB levels upregulate BACE1 promoter activity and BACE1 transcription. In the present study, when N2a Swe cells were exposed to 1% FBS (to overproduce Aβ and C99), P-JAK2 and P-STAT3 expressions were significantly elevated at 3 hr, and these up-regulations were suppressed by taxifolin, cilostazol, and by AG490 (a JAK2 inhibitor), indicating P-STAT3 expression is increased via the mediation of P-JAK2 activation. The expression of P-STAT3 in cytosol was significantly elevated at 3 hr under 1% FBS and then nuclear P-STAT3 expression increased from 3 to 12 hr, and its elevation was suppressed by taxifolin plus cilostazol, which indicates a decrease in nuclear P-STAT3 levels by these drugs.

Several lines of study have revealed NF-κB inhibitors are therapeutically important for treatment of AD pathology because the inhibitors block inflammatory processes and directly inhibit the production of Aβ peptides [19] and NF-κB inhibition prevents Aβ-induced BACE1 promoter transactivation [8,29]. Herein, we assessed the impact of taxifolin on the Aβ-stimulated degradation of IκBα in cytosol and on the nuclear translocation of NF-κB. Notably, cilostazol has been reported to inhibit NF-κB activation [17,18]. Consistent with these reports, decreased cytosolic IκBα expression and increased nuclear NF-κB p65 levels in activated N2a Swe cells were totally prevented by pretreating taxifolin plus cilostazol. Furthermore, co-treatment with lower concentrations of taxifolin plus cilostazol caused striking increases in IκBα expression and decreased nuclear NF-κB p65 expression, whereas the monotherapy with same concentration could not influence. These findings were further supported by our NF-κB p65 DNA binding assay results, which showed that the increase in NF-κB activity was substantially decreased by co-treatment with lower doses of taxifolin and cilostazol. Overall, these results confirm the synergistic effect of combinatorial taxifolin plus cilostazol therapy.

Earlier, it was reported interactions between STAT3 and p300 or histone acetyltransferase (HAT) are required for STAT3-dependent transcription [30], and that the reversible acetylation of RelA regulates the duration of the nuclear activity of NF-κB [31]. After phosphorylation of its critical tyrosine residue (Tyr 705), STAT3 dimerizes due to a phosphotyrosine-SH2 domain interaction [32]. According to a report issued by Grivennikov and Karin [13], activated STAT3 interacts with RelA/p65 to recruit p300 HAT complex and causes RelA acetylation that prolongs its nuclear retention. Once dimerized, STAT transcription factors enter the nucleus where they interact with RelA/p65, and subsequently recruit p300-HAT. In addition, p300 can acetylate p65 and increase its nuclear retention, and thereby, enhance its transcriptional activity [33]. Given that STAT3 prolongs the nuclear retention of NF-κB, P-STAT3 inactivation by taxifolin plus cilostazol may decrease NF-κB activity.

Taxifolin is a dihydroquercetin flavonoid found in onions and French maritime pine bark [14]. Several studies have reported that natural flavonoids, such as, myricetin and quercetin directly inhibit BACE1 in a concentration-dependent manner and concomitantly inhibit Aβ_1–42_ levels [22]. The present study shows taxifolin, cilostazol, and quercetin all significantly diminished the mRNA and elevated protein expressions of BACE1 in activated N2a Swe cells.

NAD^+^-dependent protein deacetylase, particularly SIRT1, has attracted attention since it was reported it down-regulated the transactivation potential of the RelA/p65 subunit of NF-κB [34]. Lee et al. [20] previously reported cilostazol (3–10 μM) stimulates SIRT1 mRNA and protein expression and increases SIRT1 activity in N2a cells. Of the seven sirtuins (SIRT1-7), SIRT1 has been strongly implicated in AD, that is, loss of SIRT1 was found to be closely associated with Aβ accumulation in the brains of AD patients [35, 36]. In the present study, taxifolin, cilostazol, and resveratrol all significantly stimulated SIRT1 protein expression and SIRT1 activity. Interestingly, in N2a cells transfected with SIRT1 siRNA, both taxifolin and cilostazol failed to suppress the Aβ_1-42_-stimulated expressions of P-STAT3 and BACE1, which contrasted to that observed in cells transfected with scrambled siRNA duplex (negative control), indicating both taxifolin and cilostazol inhibit the expressions of P-STAT3 and BACE1 by up-regulating SIRT1. These results suggest BACE1 inhibition could be a valid therapeutic target in AD.

In agreement with the suggestion made by Fusco et al. [37] that CREB directly regulates SIRT1 mRNA and protein expression in neurons, Lee et al. [20] showed a decrease in CREB/P-CREB Ser133 and of SIRT1 mRNA was induced by endogenous Aβ overproduction in activated N2a Swe cells. Alternatively, when these cells were pretreated with cilostazol, P-CREB Ser133 expression was significantly elevated and the decrease in SIRT1 mRNA expression was prevented. In view of these findings, we believe it likely that the up-regulation of P-CREB Ser133 by cilostazol increases SIRT1 transcription in neurons. However, it remains to be determined how taxifolin increases SIRT1 expression.

NF-κB is a central regulator of microglial responses to proinflammatory stimuli, including LPS and cytokines [38]. A number of reports have shown LPS strongly activates microglia and induces the COX-2 gene, leading to the synthesis of PGE2 [39]. Furthermore, the expressions of iNOS and COX-2 are known to be regulated by the activation of NF-κB. Reportedly, there is one NF-κB DNA consensus sequence within COX-2 promoter, and two NF-κB DNA consensus sequences within iNOS promoter [40–42]. In the present study, when BV-2 microglia were exposed to LPS, nitrite levels (a marker of NO production) significantly increased in conjunction with the expressions of iNOS and COX2, and these increased variables were attenuated by taxifolin (3 ~100 μM) and by cilostazol (3 ~ 30 μM). In addition, we observed the reduced viability of N2a cells by Aβ_1–42_ was significantly prevented by 10 or 30 μM of taxifolin or cilostazol, respectively. In light of these results, the suppression of NF-κB by co-treatment with taxifolin and cilostazol provides a potential strategy for the management of AD by reducing neuroinflammation and Aβ generation.

Taken together, these results indicate co-treatment with taxifolin and cilostazol inhibits amyloidogenesis by suppressing P-JAK2/P-STAT3-coupled NF-κB-linked BACE1 expression via the up-regulation of SIRT1 in a synergistic manner, and suppresses proinflammatory reactions and increases cell viability. These results may provide a rationale for combinatorial therapy with taxifolin and cilostazol in clinical trials targeting AD.

## Materials and Methods

### Agents and antibodies

Anti-iNOS, anti-COX-2, anti-P-STAT3 (Tyr 705), anti-JAK2 and anti-P-JAK2 (Tyr1007/1008) antibodies were obtained from Cell Signaling (Danvers, MA). Anti-BACE1, anti-STAT3, anti-NF-κB p65, anti-IκBα, anti-SIRT1, and anti-histone H1 antibodies were purchased from Santa Cruz Biotechnology Inc. (Santa Cruz, CA). Anti-Aβ (6E10) anti-body was from Covance (Emeryville, CA). AG490 was from Tocris Bioscience (Bristol, UK). BAY11-7082, sirtinol, and quercetin were from Sigma-Aldrich (St. Louis, MO). Aβ_1–42_ peptide was purchased from AnaSpec (Fremont, CA). Taxifolin and cilostazol were donated by Otsuka Pharmaceutical Co. Ltd. (Tokushima, Japan).

### Cell culture

Mouse neuroblastoma N2a wild-type cells and N2a Swe cells were donated by Dr. Takeshi Iwatsubo (Department of Neuropathology and Neuroscience, Graduate School of Pharmaceutical Sciences, University of Tokyo) [20] and grown in DMEM medium containing 10% fetal bovine serum (FBS). To evoke endogenous Aβ overproduction, the culture medium for N2a and N2a Swe cells was switched from DMEM containing 10% fetal bovine serum (FBS) to DMEM containing 1% FBS, and cells were then cultured for 3 ~ 24 hr. BV-2 cells were obtained from the American Type Culture Collection (Rockville, Maryland) and grown in DMEM containing 10% FBS. By Western blot analysis, we confirmed the purity of BV2 cells: the cells highly expressed CD11b and CD68 proteins, indicating microglial cell line (data not shown).

### NF-κB p65 transcription factor assay

To assess nuclear NF-κB transcription levels, cell lysates were extracted using a nuclear extraction kit (Enzo Life Sciences, Farmingdale, NY). Specific DNA binding activities of NF-κB p65 in nuclear extracts were determined using NF-κB p65 transcription factor assay kit (Active Motif, Carlsbad, CA).

### Western blot analysis

Proteins (30 μg) were loaded onto 8 ~ 15% SDS-polyacrylamide electrophoresis gels, electrophoresed, and transferred to nitrocellulose membranes (Amersham Biosciences, Inc., Piscataway, NJ), which were then incubated with appropriate antibodies. Immunoreactive bands were visualized using the chemiluminescent reagent in the Supersignal West Dura Extended Duration Substrate Kit (Pierce Chemical, Rockford, IL). Signals from bands were quantified using a GS-710 calibrated imaging densitometer (Bio-Rad, Hercules, CA).

### Immunocytochemistry

Cells were cultured in cover glass-bottomed dishes, fixed with 4% paraformaldehyde, permeabilized with 0.2% Triton X-100 in PBS. Expression of NF-κB p65 was detected using anti-NF-κB p65 antibody. Cells were incubated with primary antibodies for 4 hrs, and then with Cy3-conjugated secondary antibodies for 1 hr. Fluorescent images were obtained using a confocal microscope (OLYMPUS FV-1000, Tokyo).

### STAT3 siRNA and SIRT1 siRNA transfection assays

STAT3 siRNA oligonucleotide (GenBank accession no. NM_213659.2) and SIRT1 siRNA oligonucleotide (GenBank accession no. NM_019812.1) were synthesized by Bioneer (Daejeon, Korea). siRNA molecules were transfected into cells using Lipofectamin 200 siRNA transfection reagent (Invitrogen, Carlsbad, CA), according to the manufacturer's instructions.

### Quantitative real-time reverse transcription

Total RNA was extracted from cells using TRIzol reagent (Invitrogen, San Diego, CA). BACE1 gene expression was determined by real-time PCR using a LightCycler 96 system (Roche Molecular Biochemicals, Mannheim, Germany) instrument and LightCycler DNA Master SYBR Green I (Roche) using 1 ng of reverse-transcribed total RNA. PCR was performed using 95°C for 10 min followed by 50 amplifications of 95°C for 10 s, 50°C for 10 s, and 72°C for 10 s. The following oligonucleotide primers specific for BACE1 were used: murine BACE1: 5′-GCATGATCATTGGTGGTATC-3′(sense) and 5′-CCATCTTGAGATCTTGACCA-3′(antisense). Data were analyzed using LightCycler 96 Software (Roche Molecular Biochemicals).

### NF-κB DNA binding assay

To measure NF-κB transcription factor levels in nuclear extracts, cell lysates were extracted using a nuclear extraction kit (Chemicon International, Temecula, CA). NF-κB p65 DNA binding activities were determined using a colorimetric NF-κB p65 transcription factor assay kit (Rockland Immunochemicals, Gilbertsville, PA), according to the manufacturer's instructions.

### Determination of nitric oxide (NO) production

BV2 microglial cells (1.5 × 10^5^ cells/ml) were grown in 96-well plates and then incubated with or without LPS (1 μg/ml) in the absence or presence of different concentrations of taxifolin for 24 hours. Nitrite accumulations in supernatant were assessed using a Griess assay. Briefly, 50 μl of culture supernatant was mixed with an equal volume of Griess reagent [0.1% N-(1-naphthyl)-ethylenediamine and 1% sulfanilamide in 5% phosphoric acid] and incubated at room temperature for 10 minutes. Absorbance at 540 nm was measured using a microplate reader (Thermo Electron Corporation, Marietta, OH) using sodium nitrite as a standard.

### SIRT1 deacetylation assay

The *in vitro* deacetylase assay was performed using a fluorometric SIRT1 Assay Kit (CS1040; Sigma), according to the manufacturer’s protocol. Briefly, the reaction was carried out at 37°C for 30 min and deacetylase activity was detected as a fluorescent emission at 450 nm, using an excitation wavelength of 360 nm. The fluorescence intensities of compounds at 450 nm were subtracted from initially determined assay values. The assay was performed in duplicate at least three times.

### Statistical analysis

Results are presented as means ± SEMs. One-way analysis of variance (ANOVA) followed by Tukey’s *post hoc* multiple comparisons method was used to determine the significances of intergroup differences. The student’s *t*-test was used to determine the significances of differences means of untreated and inhibitor treated cells. Statistical significance was accepted for *P* values of < 0.05.

## Abbreviations

AD: Alzheimer’s disease
BACE1: β-site APP cleaving enzyme 1
C99: C-terminal fragment β
JAK2: Janus kinase 2
STAT3: signal transducer and activator of transcription 3
